# Assessment of current mass spectrometric workflows for the quantification of low abundant proteins and phosphorylation sites

**DOI:** 10.1016/j.dib.2015.08.015

**Published:** 2015-09-04

**Authors:** Manuel Bauer, Erik Ahrné, Anna P. Baron, Timo Glatter, Luca L. Fava, Anna Santamaria, Erich A. Nigg, Alexander Schmidt

**Affiliations:** a Biozentrum, University of Basel, Klingelbergstrasse 50/70, CH-4056 Basel, Switzerland; b Division of Developmental Immunology Biocenter, Innsbruck Medical University, Innraion 80, 6020 Innsbruck, Austria; c Cell Cycle and Mitosis Laboratory, Research unit in Biomedicine and Translational Oncology, Vall Hebron Institute of Research, Psg. Vall d’Hebron 119-129, 08035 Barcelona, Spain

## Abstract

The data described here provide a systematic performance evaluation of popular data-dependent (DDA) and independent (DIA) mass spectrometric (MS) workflows currently used in quantitative proteomics. We assessed the limits of identification, quantification and detection for each method by analyzing a dilution series of 20 unmodified and 10 phosphorylated synthetic heavy labeled reference peptides, respectively, covering six orders of magnitude in peptide concentration with and without a complex human cell digest background. We found that all methods performed very similarly in the absence of background proteins, however, when analyzing whole cell lysates, targeted methods were at least 5–10 times more sensitive than directed or DDA methods. In particular, higher stage fragmentation (MS3) of the neutral loss peak using a linear ion trap increased dynamic quantification range of some phosphopeptides up to 100-fold. We illustrate the power of this targeted MS3 approach for phosphopeptide monitoring by successfully quantifying 9 phosphorylation sites of the kinetochore and spindle assembly checkpoint component Mad1 over different cell cycle states from non-enriched pull-down samples. The data are associated to the research article ‘Evaluation of data-dependent and data-independent mass spectrometric workflows for sensitive quantification of proteins and phosphorylation sites׳ (Bauer et al., 2014) [1]. The mass spectrometry and the analysis dataset have been deposited to the ProteomeXchange Consortium (http://proteomecentral.proteomexchange.org) via the PRIDE partner repository with the dataset identifier PXD000964.

## Specifications table

**Table:** 

Subject area	Biology, chemistry
More specific subject area	Phosphoproteomics and mass spectrometry (MS)
Type of data	MS-data, Tab-delimited and Microsoft Excel tables
How data was acquired	Easy-nLC liquid chromatography system coupled to either a LTQ Orbitrap Velos or a TSQ Vantage mass spectrometer (all Thermo Scientific)
Data format	Raw (.raw), mgf peak lists (.mgf), transition lists and Skyline processed files (.csv and.xlsx)
Experimental factors	Sensitivity assessment of different LC–MS approaches using dilution series experiments and application of the most sensitive MS method to quantify MAD1 phosphorylation sites during cell cycle.
Experimental features	Different dilution series of peptides were analyzed using one dimensional liquid chromatography separation and different data-dependent and independent mass spectrometry workflows to assess their limits of quantification. The most sensitive method was applied to monitor phosphorylation site changes in immunopurified MAD1 across different cell cycle stages using thymidine and nocodazole treatment.
Data source location	Basel, Switzerland
Data accessibility	The data are available via ProteomeXchange with identifier PXD000964 http://proteomecentral.proteomexchange.org/cgi/GetDataset?ID=PXD000964

## Value of the data

• Assessment of detection and quantification limits for data-dependent, directed and targeted mass spectrometric workflows
• Individual MS workflow evaluation for unmodified and phosphorylated peptides
• The ability to monitor low abundant peptides and phosphorylation sites is demonstrated by quantitation of phosphorylation sites from immunopurified MAD1 protein during the cell cycle without the need for phosphopeptide enrichment steps.
• Data can be employed to evaluate and improve identification and quantification capability of proteomics software tools to monitor low abundant peptides/proteins and protein modifications.

## 1 Data, experimental design, materials and methods

### 1.1 Experimental design

**Fig. 1:**
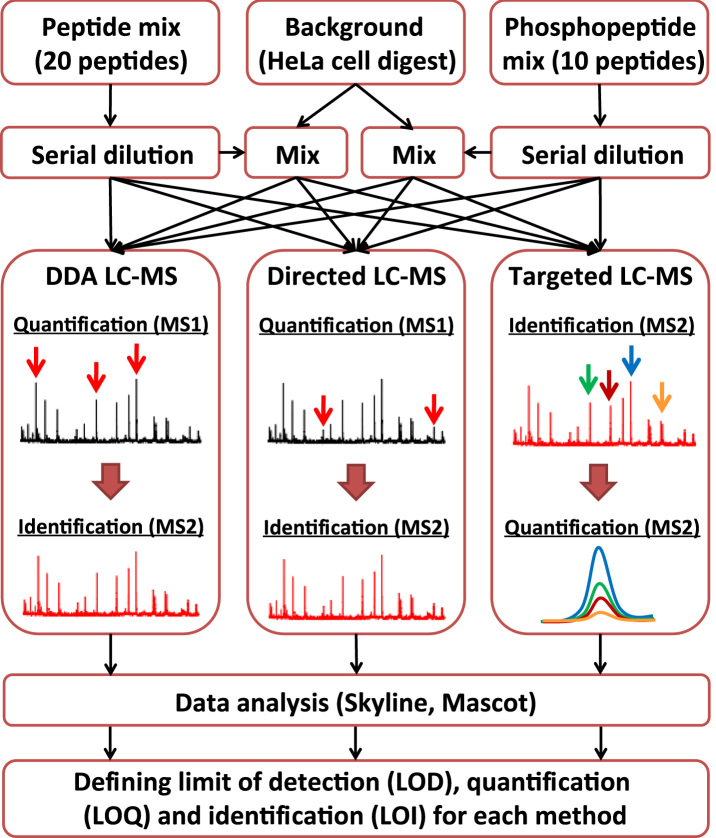
Experimental setup for the determination of quantification and detection limits for data-dependent and independent quantitative mass spectrometry workflows.

The general aim of this study was to assess the capabilities of recently established data-dependent (DDA) and independent (DIA) LC–MS strategies [2] in terms of sensitivity and linear relative quantification range for a variety of different peptides in the presence and absence of a complex analytical background (see Fig. 1). Therefore, we prepared serial dilutions of two different peptide mixtures consisting of 20 unmodified and 10 phosphorylated chemically synthesized heavy peptides covering a concentration range of 6 and 5 orders, respectively (referred to as neat samples). To assess analytical performance under a more realistic scenario, we prepared the same dilution series with a complex human digest spiked into each sample (referred to as complex samples). This allowed us to precisely determine the impact of the analytical background on limit of detection (LOD), quantification (LOQ) and identification (LOI) for each peptide and MS-approach applied in this study. The four dilution series were analyzed in duplicate using the following DDA and DIA MS-approaches. (i) In DDA, only peptides of the highest intensities in the acquired survey scans are selected for MS-sequencing while many other peptides of sufficient intensity for identification pass through the instrument to remain unidentified. (ii) Directed LC–MS/MS strategy (also termed inclusion mass list driven, INL) that attempts to overcome this limitation by directing MS-sequencing to the precursors of interest independent of their MS-intensities using an inclusion mass list. Albeit several thousands of peptides can be analyzed by this approach, a drawback is the necessity to detect the precursor ions in the MS1 survey scans to trigger fragmentation. (iii) Targeted LC–MS/MS methods (selected reaction monitoring (SRM) and pseudo-selected reaction monitoring (pSRM, like SRM, but a full MS/MS-scan is acquired) that directly fragment selected peptide ions and use the corresponding fragment ions for identification and quantification. We analyzed each sample by SRM using a triple quadrupole (QqQ) MS and by pSRM using collision induced dissociation (CID, ion detection in the linear ion trap (LIT)) based fragmentation. For phosphopeptide analysis, we additionally carried out higher energy collision dissociation (HCD, ion detection in the orbitrap) and neutral loss MS2/MS3 CID fragmentation in the LIT. All peptides were quantified using Skyline [3,4] and fragment spectra were identified by database searching. Finally, we generated dilution profile correlations for each peptide and MS-method and applied an established algorithm [3,4] to determine linear quantification ranges as well as identification, quantification and detection limits.

### 1.2 Cell culture and sample preparation of human cell digest

HeLa S3 were cultured in Dulbecco׳s modified Eagle׳s medium (DMEM, Invitrogen, Carlsbad, CA) supplemented with 10% heat-inactivated fetal calf serum (FCS) and penicillin–streptomycin (100 IU/ml and 100 μg/ml, respectively, GIBCO) at 37 °C in a 5% CO_2_ atmosphere in a humidified incubator. 10^7^ cells were collected by centrifugation and cell pellets washed twice with PBS. Cells were lysed in 200 µl lysis buffer (8 M Urea, 0.1% RapiGest, 0.1 M ammoniumbicarbonate) using strong ultra-sonication and total protein concentration determined by BCA assay (Thermo Scientific) according to the manufacturer׳s instructions. Then, proteins were reduced with 5 mM TCEP for 60 min at 37 °C and alkylated with 10 mM iodoacetamide for 30 min in the dark at 25 °C. After quenching the reactions with 12 mM N-acetyl-cysteine, protein samples were digested by incubation with sequencing-grade Lys-C (1/200, w/w; Wako) for 4 h at 37 °C. Samples were diluted 1:4 with 0.1 M ammoniumbicarbonate buffer to reduce urea concentration to 1.6 M and digestion continued by adding modified trypsin (1/50, w/w; Promega, Madison, Wisconsin) over night at 37 °C. Subsequently, peptides were desalted on C18 reversed-phase columns according to the manufacturer׳s instructions (SEP-PAK Vac 3cc 500 mg, Waters), dried under vacuum and stored at −80 °C until further use.

### 1.3 Generation of serial dilution mixtures

We took advantage of an ongoing parallel study aiming at the absolute quantification of centrosomal proteins and employed the chemically synthesized 20 heavy labeled reference peptides (AQUA grade, Thermo Scientific, Table S1) as spike in standards for our systematic quantitative evaluation of different MS approaches. In this study, for each of the 10 centrosomal proteins of interest the two full tryptic peptides with the highest MS-intensities lacking any missed cleavages were selected as reference peptides. Subsequently, a mixture comprising equal concentrations of all peptides was prepared and a dilution series generated using 10-fold steps starting from 0.5 pmol/µl to 0.5 amol/µl. To minimize peptide losses during pipetting and storage, low binding tips (Axygen) and glass vials (VWR International) were applied for all sample preparation steps. Next, the same dilution series was prepared adding the human cell digest sample at a concentration of 0.5 ug/µl to all samples.

In a second dilution experiment, we employed a standard mixture containing 10 singly and doubly phosphorylated peptides in equal amounts (MS PhosphoMix 1 Heavy, Sigma-Aldrich, Table S1) and prepared the same two dilution series (with and without a human cell digest) as described above, starting from 50 fmol/µl to 0.5 amol/µl. 2 µl of each sample were subjected to LC-MS analysis.

### 1.4 Mass spectrometric analysis

#### 1.4.1 Data-dependent acquisition (DDA) LC–MS/MS

Peptides were separated on a RP-LC column (75 μm×20 cm) packed in-house with C18 resin (Magic C18 AQ 3 μm; Michrom BioResources, Auburn, CA, USA) using a linear gradient from 95% solvent A (98% water, 2% acetonitrile, 0.15% formic acid) and 5% solvent B (98% acetonitrile, 2% water, 0.15% formic acid) to 30% solvent B over 40 min at a flow rate of 0.2 μl/min. Each survey scan acquired in the Orbitrap at 60,000 FWHM was followed by 20 MS/MS scans of the most intense precursor ions in the linear ion trap with enabled dynamic exclusion for 20 s. Charge state screening was employed to select for ions with at least two charges and rejecting ions with undetermined charge state. The normalized collision energy was set to 32% and one microscan was acquired for each spectrum. Collision induced dissociation was triggered when the precursor exceeded 100 ion counts. The ion accumulation time was set to 300 ms (MS) and 50 ms (MS/MS). All samples were measured in triplicates. Phosphopeptide analysis was carried out as described above with the following modification: each survey scan was followed by 10 MS/MS scans of the most intense precursor ions in the linear ion trap with enabled multistage activation.

#### 1.4.2 Directed (INL) LC–MS/MS

For directed LC–MS/MS two inclusion mass lists comprising the calculated ion masses of the observed precursor ions of either 20 unmodified (Table S2) or 10 phosphorylated (Table S3) peptides were generated and imported as mass lists to the instrument software. LC–MS analysis was carried out using the same settings as for DDA analysis with a few modifications; mono-isotopic precursor selection was disabled and peaks with unassigned charge states were not rejected. This helped to trigger more MS-sequencing attempts. Furthermore, ion accumulation time for MS2 scans was set to 100 ms.

#### 1.4.3 Pseudo-selected reaction monitoring (pSRM) LC–MS/MS

PRM was carried out in the linear ion trap (LIT) (CID) and Orbitrap (HCD), respectively. For both experiments, the peptides and their modifications were imported into the Skyline software (version 2.4) (https://skyline.gs.washington.edu/labkey/wiki/home/software/Skyline/page.view?name=default) [4,5]. The precursor ion masses were automatically calculated and the masses of all observed precursor ions exported as an instrument method file (Tables S2 and S3). For pSRM-CID-MS2 ion accumulation time was set to 10 ms and the mass selection window was set to 1 Da. The collision energy was set to 35% and activation time was 10 ms. Fragment ions were scanned from the lowest possible *m*/*z* to 2000 Th. For pSRM-HCD, MS2 spectra were acquired at a resolution of 7500 (FWHM at 400 *m*/*z*), ion accumulation time was 50 ms, mass selection window was set to 2 Da, collision energy of 35%, activation time of 100 ms and the measured mass range was from 100 Th to 2 times the precursor mass. Additionally, corresponding charge states were set in the instrument HCD fragmentation method. For pSRM-CID-MS3 analysis, the neutral loss masses were manually calculated and added to the pSRM-CID-MS2 instrument method. Here, to increase sensitivity, the mass selection window was set to 2 Da for MS2/MS3 ion isolation and an ion accumulation time of 50 ms was applied.

#### 1.4.4 SRM LC–MS/MS (on triple quadrupole instrument)

Data derived from a spectral library generated based on acquired HCD spectra of the standard peptide mix from the PRM-HCD experiment were imported into the Skyline program (version 2.4) to extract the corresponding fragment ion masses and precursor ion masses (transitions). After collision energy optimization, the five most suited transitions per peptide were selected and traced on a triple quadrupole mass spectrometer (QqQ, TSQ Vantage, Thermo Scientific) connected to an electrospray nano ion source and Easy nano-LC system (both Thermo Scientific) using the same setting as used for DDA LC–MS analysis. Cycle time was set to 2 s resulting in a dwell time of 20 ms per transition. The transition lists with optimized collision energies comprising the 20 unmodified and 10 phosphopeptides are provided as supplemental Tables S4 and S5, respectively.

### 1.5 Data processing

#### 1.5.1 Peptide quantification/determination of quantification limits

All raw files were loaded into the Skyline software tool (version 2.4) to generate extracted ion chromatograms of the precursor (up to 5) or fragment (up to 10) ions. The mass windows were adjusted to the resolution applied in the corresponding MS method. For PRM-CID methods, a mass window of 0.4 Da was applied. To make the PRM-CID-MS3 data files readable for the Skyline software, we converted the raw files to mzXML format using MM-conversion tool (version 3.9, www.massmatrix.org) and replaced the neutral loss masses used for MS3 by the corresponding original precursor ion masses using an in-house Perl script (available upon request). All integrated peak/transitions were manually inspected and corrected or removed, if required. The integrated and quantified peak/transitions obtained for the different methods and samples are listed in Tables S6–14. Finally, we generated dilution profile correlations and applied an established algorithm [3] to determine LOQ and LOD values as well as linear correlations (Pearson׳s correlation coefficient (*R*^2^) from highest concentration to LOQ) for each MS-method and peptide analyzed.

#### 1.5.2 Determination of identification limits

All raw files acquired by DDA, INL and pSRM for the dilution curve samples of unmodified peptides were converted to mgf-format using the MM-conversion tool (version 3.9, www.massmatrix.org) and searched against a decoy (consisting of forward and reverse protein sequences) human SwissProt database (download date 16.05.12) containing known contaminants resulting in a total of 41,250 protein sequences using Mascot (Matrix Science, version 2.4). The search parameters were set as follows; full tryptic specificity was required (cleavage after lysine or arginine residues unless followed by proline), up to two missed cleavages were allowed, carbamidomethyl (C) was set as fixed modification, oxidation (M), Label: 13C(6)15N(2) (K) and Label: 13C(6)15N(4) (R) as variable modification, 10 ppm precursor mass tolerance and 0.6 (0.02) Da fragment mass tolerance for CID (HCD) tandem mass spectra. After importing the data to the Scaffold software (http://www.proteomesoftware.com, version 4.2.1) the FDR rate was set to <1% for MS/MS-spectra identifications by the Scaffold Local FDR algorithm based in the number of decoy hits. All identified MS/MS-spectra in the dilution curve experiment for DDA, INL and pSRM are available as supplement data (Tables S15–S17).

### 1.6 Monitoring of Mad1 phosphorylation sites

#### 1.6.1 Cell culture, synchronization and kinase inhibitors

HeLa S3 were cultured as described above. Cell cycle arrest in S-phase was induced by thymidine (2 mM, Sigma-Aldrich) treatment for 24 h. For MS analysis of mitotic cell cycle stages, cells were released from thymidine and arrested in mitosis before harvesting. Mitotic arrest in prometaphase was induced by Nocodazole (0.5 μg/mL, Sigma-Aldrich) treatment for 14 h after thymidine release. Mitotic cells were collected by mitotic shake-off. Mitotic arrest in metaphase was induced by addition of the proteasome inhibitor MG132 (10 μM, Calbiochem) for 2 h, 10 h after thymidine release.

#### 1.6.2 Cell extracts and immunoprecipitations

For preparing extracts, HeLa S3 cells were washed once with ice-cold PBS, and resuspended in ice-cold lysis buffer (20 mM Tris, pH 7.4, 150 mM NaCl, 0.5% IGEPAL CA-630, 1 mM DTT, 30 μg/ml RNAse, 30 μg/ml DNAse, protease inhibitor cocktail (Roche, 1 EDTA- free tablet for 10 ml lysis buffer) and phosphatase inhibitors cocktail (cocktails 2 and 3; Sigma- Aldrich) and incubated for 30 min on ice. After cell lysis, suspensions were cleared by centrifugation at 14,000 rpm for 15 min. Immuno-purification of endogenous Mad1 was performed using 50 μl of solid Affi-Prep protein G matrix beads (Bio-Rad Laboratories) chemically cross-linked to 1 μg/μl of antibody against 1–2 mg of clarified cell lysate for 2 h at 4 °C. Afterwards the resin was washed with lysis buffer followed by washing with HNN buffer (50 mM Hepes pH7.5, 150 mM NaCl, 5 mM EDTA, 50 mM NaF). Proteins were eluted with 100 mM glycine pH 2.8, neutralized by the addition of Tris buffer (pH 8,0), reduced, alkylated, enzymatically cleaved and prepared for MS analysis as described above. For generating a comprehensive phosphorylation site map of Mad1, immune-purified proteins obtained from 10 (S-phase) and 15 (prometaphase and metaphase) 15 cm dishes were pooled, split in two aliquots and subjected to the two different phosphopeptide enrichment strategies described below. For monitoring of Mad1 phosphorylation sites, sufficient protein amounts could be obtained from 1 (S-phase) and 2 (prometaphase and metaphase) 15 cm dishes. After sample preparation, the peptide samples were dissolved in 40 µl of 0.1% formic acid containing 125 fmol/μl of each heavy labeled phosphopeptides.

#### 1.6.3 Phosphopeptide enrichment (TIO_2_)

30 μl of titanium dioxide beads (100 mg/ml, Titansphere, GL Sciences Inc, Japan) were placed on self-made GELoader tips (Eppendorf) plugged with a piece of C8 material (Empore, 3M, 3M Empore C8 and C18 disks, 2214-C8, Bioanalytical Technologies,St. Paul, MN). The columns were washed with water (HPLC grade, Sigma-Aldrich), methanol and a solution of 80% ACN (Acetonitrile), 2.5% TFA (Trifluoroacetic acid), saturated with phthalic acid. Digested and dried peptides were reconstituted in 80% ACN, 2.5% TFA, saturated with phtalic acid and loaded on the micro-columns. To allow maximal binding of phosphorylated peptides to the titanium dioxide beads the peptide-bead mixture was incubated for 10 min, then slowly passed through and applied for two additional times. The micro-columns were subsequently washed with a mixture of 80% ACN and 2.5% TFA, saturated with phthalic acid, a mixture of 80% ACN, 20% water and 0.1% TFA and finally with 0.1% TFA. Phosphorylated peptides bound on the TiO_2_ were eluted with 0.3 M ammonium hydroxide solution. Phosphopeptide enriched eluates were immediately acidified with 2 M HCl and 5% TFA, desalted and purified on C18 Microspin columns (Harvard Apparatus) and dried in a SpeedVac concentrator.

#### 1.6.4 Phosphopeptide enrichment (IMAC)

PHOS-Select™ Iron Affinity Gel beads (Sigma-Aldrich) and dried peptides were re-suspended in 30% acetonitrile/250 mM ethanol. IMAC beads and peptides were shaked at room temperature at 1400 rpm for 2 h. Subsequently samples were loaded three times in a constricted GELoader tip and washed four times with 30% acetonitrile/250 mM ethanol. Phosphorylated peptides were eluted using 50 mM Na_2_HPO_4_/NH_3_ (pH 10.0), acidified with 100% ethanol and 10% TFA (pH<3.5), desalted and passed to LC–MS/MS analysis.

#### 1.6.5 Generation of a Mad1 phosphorylation site catalog from phosphopeptide enriched samples

1 µg of total phosphopeptides were subjected to DDA LC–MS/MS using HCD and CID with enabled multistage activation fragmentation as specified above. Acquired raw files were database searched using Mascot and Scaffold software as described above with the following parameter modification: oxidation (M), Label: 13C(6)15N(2) (K), Label: 13C(6)15N(4) (R) and phosphorylation (S, T, Y) were set as variable modification. The identified proteins, peptides and MS/MS-spectra (Tables S18–S20) were filtered to a FDR of 1% according to the Scaffold Local FDR algorithm based in the number of decoy hits. A list of all MS/MS-spectra assigned to Mad1 phosphopeptides is shown in Table S21 and a summary list comprising all identified Mad1 phosphorylation sites is illustrated in Table 1. Protein probabilities were assigned by the Protein Prophet program. Proteins that contained similar peptides and could not be differentiated based on MS/MS analysis alone were grouped to satisfy the principles of parsimony. Proteins sharing significant peptide evidence were grouped into clusters. The location of the phosphorylated residues was automatically assigned by MASCOT (score>10).

#### 1.6.6 Mad 1 phosphorylation site monitoring

Heavy labeled reference peptides were synthesized for all identified phosphopeptides of the Mad1 proteins. For precise quantitation of the single serine phosphorylation sites at positions S484, S485, S486 and S490, we ordered all possible mono-phosphorylated versions of the corresponding heavy reference peptide (SQSSSAEQSFLFSR). All different phosphopeptide sequences and modifications together with the identified MS/MS-spectra from the previous phosphorylation catalog experiment were imported into the Skyline software (Version 2.4) to set up a pSRM method to monitor all phosphopeptides. In an initial analysis, we carried out MS2 and MS3 based PRM analysis to select for the best transitions for each peptide. Due to their higher selectivity and lower noise levels, transitions originating from MS3 scans were preferred, if available. Phosphopeptides were quantified using the Skyline tool and the same parameters as described above. The quantitative results including normalization, ratio determination and statistical analysis are summarized in Tables S22 and S23.

For the determination of phosphorylation site stoichiometries and to compensate variations in Mad1 concentrations, additional label-free quantification experiments of the same samples were carried out. 1ug of peptides were subjected to DDA LC–MS/MS analysis using CID with enabled multistage activation fragmentation and the same LC and MS settings as specified above. The LC–MS Progenesis software (Version 4.1.4832.42146) in combination with the Mascot database search tool (Version 2.4) was employed to identify and quantify unmodified and modified Mad1 peptides using the same database search parameters for phosphopeptides as described above. Importantly, the Progenesis software was set that only non-conflicting peptides with specific sequences for single proteins in the database were employed for quantification. The results were further statically validated by our in-house software tool SafeQuant [6] (available upon request). All identified and quantified peptides are listed in Table S24. Differences in Mad1 concentrations in the samples were normalized using the sum of all MS-intensities generated from Mad1 peptides. The corresponding normalization factors using the first sample as base are displayed in Table S23. The data file names of LC–MS runs of all samples analyzed in this study are shown in Table S25.

## Figures and Tables

**Table 1:** Phosphopeptides identified for the protein Mad1.

Best ion score a	Sequence	Precursor ion charge	Phosphosite position	Phosphosite reported previously^b^	Putative upstream kinase(s)^c^
27.71	***S***LNNFISQR	2+	S16	Yes	Mps1, Plk1
34.77	IQELQA***S***QEAR	2+	S214	Yes	Mps1, Plk1, ATM kinase
33.02	DLEQKL***S***LQEQDAAIVK	3+	S233	No	
41.78	L***S***LQEQDAAIVK	2+	S233	No	
46.69	AILGSYDSELTPAEY***S***PQLTR	3+	S428	Yes	Mps1, Cdk1
54.81	AILGSYDSELTPAEY***S***PQLTR	2+	S428	Yes	Mps1, Cdk1
83.7	SQ***S***SSAEQSFLFSR	2+	S484	No	
56.79	SQS***S***SAEQSFLFSR	2+	S485	No	
68.94	SQSS***S***AEQSFLFSR	2+	S486	No	
72.63	SQSSSAEQ***S***FLFSR	2+	S490	Yes	Mps1, Plk1
31.74	SQSSSAEQSFLF***S***REEADTLR	3+	S494	No	
28.07	EEAD***T***LR	2+	T500	No	
21.74	LKVEELEGER***S***R	3+	S513	Yes	Mps1, (Plk1)
25.55	LKVEELEGER***S***R	2+	S513	Yes	Mps1, (Plk1)
24.27	VEELEGER***S***R	2+	S513	Yes	Mps1, (Plk1)
22.61	ALQGDYDQ***S***R	2+	S538	No	Mps1, (Plk1)
25.25	LREDH***S***QLQAECER	3+	S562	Yes	Mps1, (Plk1)

a Best ion score determined by the Mascot search engine. A detailed list of all identified MS/MS-spectra is shown in Tables S20 and S21.

b Adopted from www.phosphosite.org (20.05.14).

c Predicted consensus motif for Mps1, Plk1, Cdk1 and ATM/ATR kinase.
